# Clinical profile of patients having pulmonary tuberculosis and renal amyloidosis

**DOI:** 10.4103/0970-2113.48896

**Published:** 2009

**Authors:** Ramakant Dixit, Rakesh Gupta, Lokendra Dave, Nishi Prasad, Sidharth Sharma

**Affiliations:** *Department of Pulmonary Medicine, JLN Medical College, Ajmer, India*; 1 *Department of Pathology, JLN Medical College, Ajmer, India*; 2 *Department of Medicine, Gandhi Medical College, Bhopal, India*

**Keywords:** Tuberculosis, amyloidosis, complication, disease duration

## Abstract

**Objectives:**

This study was planned to define the clinical profile of pulmonary tuberculosis (PTB) patients having renal amyloidosis, to identify the factors responsible for development of amyloidosis, to detect the time period between onset of amyloidosis and PTB, and analyze clinical features of amyloidosis in PTB patients for early diagnosis and timely assessment.

**Materials and Methods:**

Patients of PTB having pedal edema, proteinuria, and grossly diseased kidneys on ultrasound abdomen were subjected to renal biopsy and appropriate biochemical investigations. Clinical profile of biopsy proven amyloidosis cases was analyzed.

**Results:**

There were 43 patients (32 males, 11 females, age range 20–65 years) having PTB with pedal edema, proteinuria, and renal medical disease on abdominal ultrasound where amyloidosis was confirmed by renal biopsy. The total duration of illness ranged from two months to seven years (mean 2.25 years) and was less than five years in 93% patients. All patients had significant proteinuria. Nephrotic syndrome was seen in 23, hypertension in 19, hypoalbuminemia in 33, hypercholesterolemia in 29, and deranged renal functions in 32 patients. Ninety percent patients had moderate to far advanced pulmonary lesions on chest radiography with smear positivity in 21 patients.

**Conclusions:**

Renal amyloidosis is an important complication of PTB and should be suspected clinically in patients presenting with a triad of pedal edema, proteinuria, and medical renal disease on ultrasound. Contrary to general belief, renal amyloidosis may occur in PTB patients having disease for relatively shorter duration, and even if adequately treated.

## INTRODUCTION

Amyloidosis is a potentially life-threatening disorder caused by deposition of insoluble fibrilar proteins in various tissues, which commonly results in organ dysfunction or failure.^1^ Amyloidosis of the kidneys is most common, and most serious because of its ill effects on renal functions. Although there are many different types of amyloidosis, the commonest form worldwide is that which occurs secondary to chronic inflammation, in which amyloid A (AA) fibrils are derived from high circulating concentration of the acute-phase protein serum amyloid A (SAA).^2^ Rheumatoid arthritis is currently the most frequent underlying inflammatory disease in developed countries, in contrast to developing countries, where tuberculosis (TB) is still the commonest underlying cause for renal amyloidosis.^3^

The diagnosis of amyloidosis is suspected on the basis of clinical features and is established by obtaining appropriate tissue biopsy and demonstrating amyloid using appropriate stains. The disease is present for a considerable time before becoming clinically manifest. In TB, pedal edema may have other causes including anemia and malnutrition. This may result in missing amyloidosis in patients with TB. No detailed studies are available on the clinical profile of pulmonary TB (PTB) patients having renal amyloidosis in our country. In this paper, the authors present their observation in 43 patients having both diseases.

## MATERIALS AND METHODS

### Study population

Adult patients of PTB attending our department with subsequent diagnosis of renal amyloidosis constituted study population.

### Inclusion criteria

The study included patients of PTB having triad of pedal edema with or without anasarca, proteinuria, and grossly diseased kidneys on ultrasonography (USG), along with a renal biopsy report showing amyloidosis.

### Exclusion criteria

These included: (a) Patients of PTB lacking any one of the three features – pedal edema, proteinuria, and abnormal kidneys on USG. (b) Patients of PTB with above triad but having renal biopsy report other than renal amyloidosis. (c) Patients having diabetes mellitus or HIV infection.

### Investigations

The diagnosis of PTB was made by sputum examination of three samples and chest radiograph. Apart from routine investigations such as hemoglobin, blood counts, erythrocyte sedimentation rate, urine examination, etc, patients of PTB with pedal edema were further subjected to liver function tests, renal function tests, USG of abdomen and pelvic organs, blood sugar, HIV serology, etc. Other investigations also included estimation of 24-hour urinary protein, serum total proteins, and serum cholesterol levels.

### Biopsy and histopathology

Patients having pedal edema with or without anasarca and proteinuria with grossly diseased kidneys on USG abdomen were subjected to percutaneous renal biopsy from the lower pole of kidneys (previously marked by USG) using Vim-Silverman or Trucut needle. Biopsy sections were stained with hemotoxylin and eosin, and periodic acid-Schiff, and examined under light microscopy. The diagnosis of amyloidosis was confirmed by the presence classical green birefringence in Congo red stained sections viewed under polarized light, and by the typical fluorescence with thioflavine T. In order to differentiate primary from secondary variety of amyloidosis, the deposits were treated with potassium permanganate before Congo red staining. In secondary amyloidosis, the green birefringence seen under polarized light gets abolished.

### Outcome and endpoints

In biopsy proven cases of renal amyloidosis with PTB, interval between the onset of the pulmonary disease and the earliest clinical evidence of renal amyloidosis was estimated. The first detection of protein in urine, or appearance of pedal edema, was taken as evidence of renal involvement by amyloidosis. All such patients were analyzed for clinical presentation, history of anti-TB treatment, extent of pulmonary disease, activity of TB, extent of proteinuria, and other biochemical profiles.

## RESULTS

**Figure 1:**
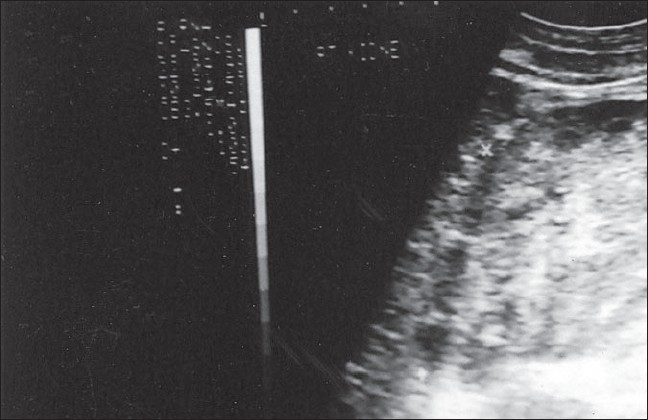
Ultrasound abdomen showing medical renal disease in form of enlarged bright kidneys with poor corticomedullary differentiation

**Figure 2:**
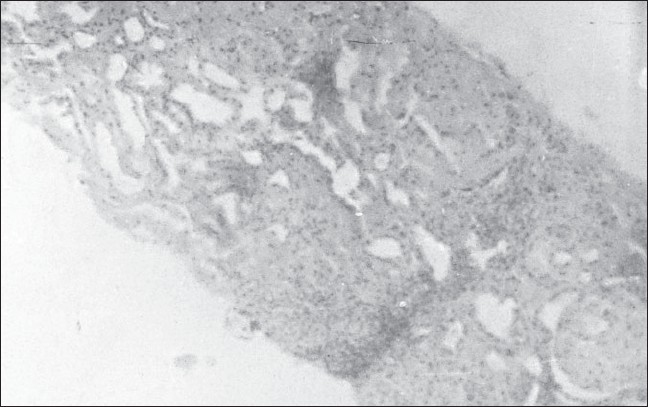
Photomicrograph of renal biopsy showing amyloid deposits in kidney tissue (H and E, ×100)

Sixty eight cases of PTB were found to have pedal edema, proteinuria, and grossly diseased kidneys on ultrasound abdomen [Figure 1]. All these patients were subjected to renal biopsy. Renal amyloidosis was confirmed in 43 patients, and these constituted the final study population [Figure 2]. The biopsy report was skeletal muscle and/or fat tissue in nineteen, blood clot in five, and cloudy swelling in one patient each.

Table 1 describes the clinical features of PTB patients with renal amyloidosis. There were 32 males and 11 females with mean age 38.48 years (age range 20–65 years). All 43 had pedal edema and 10% of them had additional generalized swelling with anasarca. Sixty percent patients were febrile and 27% had abdominal pain and distension. Cough was the commonest respiratory symptom present in all patients, followed by breathlessness (51.1%) and chest pain (27.9%). None of the patients with proven amyloidosis had any urinary symptoms.

**Table 1:** Clinical profile of patients having pulmonary tuberculosis and renal amyloidosis

Patients characteristics	Number of patients* (%)(N = 43)
Age (years)	
20–39	29 (67.4)
40–59	12 (27.9)
>60	2 (4.6)
Sex	
Male	32 (74.4)
Female	11 (25.5)
Clinical symptoms	
Constitutional	
Fever	26 (60.4)
Pedal edema alone	43 (100)
Generalized swelling	10 (23.2)
Pain and/or distension abdomen	2 (27.9)
Others	19 (44.1)
Respiratory	
Cough	43 (100)
Breathlessness	22 (51.1)
Chest pain	12 (27.9)
Hemoptysis	5 (11.6)
Time period between onset of PTB and first evidence of renal amyloidosis (years)	
1–3	29 (67.4)
3–5	11 (25.5)
<5	3 (6.9)
History of antituberculosis therapy	
Nil	9 (20.9)
Adequate	15 (34.8)
Inadequate	19 (44.1)
Radiological extent of pulmonary disease	
Minimal	4 (9.3)
Moderately advanced	18 (41.8)
Far advanced	21 (48.8)
Ultrasound findings	
Bilateral renal medical disease	43 (100)
Hepatomegaly ± spleenomegaly	24 (55.8)
Ascites	20 (46.5)
Cholicystitis ± cholithiasis	7 (16.2)

The time interval between diagnosis of PTB and the first evidence of renal amyloidosis varied from two months to seven years, with a mean of only 2.25 years or 27.1 months. The median interval between onset of symptoms and evidence of amyloid was 24 months.

Hypertension was noted in nineteen (44%) patients and it was moderately severe in six. Hepatomegaly in the absence of raised jugular venous pressure was detected in 24 (55.8%) patients and the spleen was palpable in 13 (30%) patients. About one-fourth patients showed ascites on clinical examination.

Proteinuria of varying degrees was present in all patients. It was nephrotic (>3 g/24 hours) in 23 (53.4%) patients, and 15 of them had a 24-hour proteinuria of >10 g. Microscopic hematuria and pyuria were noted in five and seven patients, respectively. Thirty three (76.7%) patients had hypoalbuminemia, and serum cholesterol was elevated in 29 (67.4%) patients (range 300–500 mg). Blood urea >50 mg/dl and serum creatinine >1.3 mg/dl were observed in 32 (74.4%) patients [Table 2].

**Table 2:** Laboratory profile of patients having pulmonary tuberculosis and renal amyloidosis

Parameters	Number of patients (%)
Anemia	27 (62.7)
Leukocytosis	8 (18.6)
Sputum for acid fast-bacilli	
Positive	21 (48.8)
Negative	22 (51.1)
Proteinuria	
4+	9 (20.9)
3+	11 (25.5)
2+	14 (32.2)
1+	9 (20.9)
Nil	-
Hypoalbuminemia	33 (76.7)
Reversal of albumin/globulin ratio	10 (23.2)
Azotemia	32 (74.4)
Hypercholesterolemia	29 (67.4)

Ninety patients had moderate to far advanced pulmonary lesions on chest X-ray. Sputum for acid fast bacilli was positive in 21 cases (eight new sputum smear positive cases, eight relapse cases, and five defaulter cases), and negative in 22 cases (15 cases with fibrotic lesions on chest X-ray and past history of adequate anti-TB treatment, one ‘new’ sputum smear negative case, and six others – smear negative but radiologically active disease cases with history of inadequate anti-TB treatment previously).

## DISCUSSION

Secondary renal amyloidosis is associated with a diverse range of disorders that usually includes chronic inflammatory disease or infectious diseases such as rheumatoid arthritis, osteomyelitis, TB, chronic bronchiectasis, empyema, ulcerative colitis, carcinomas (most commonly renal cell carcinoma), etc; and most recently in drug abusers, chronic skin infections, transmissible spongiform encephalopathies, Alzheimer's disease, and type-2 diabetes mellitus.^4^,^5^ In these conditions, proinflammatory mediators/cytokines such as interleukin-1, tumor necrosis factor alpha, and interleukin-6, stimulate the synthesis of SAA in liver and other sites, that subsequently accumulates in renal tissue.^6^ It is also to be noted that not all patients with chronic inflammatory disorders develop AA amyloidosis, and other factors such as genetic or environmental influences, specific properties of the precursor protein, macrophage activity, and the presence of ‘amyloid enhancing factor’ beside local tissue factors also play a role in amyloid fibril accumulation.^7^

Considerable variations are found in published accounts concerning the relative incidence of the various diseases associated with secondary amyloidosis. In Western countries, TB was the most important cause accounting for 50–80% of secondary causes of amyloidosis.^8^–^10^ However, rheumatoid arthritis is the common cause these days. The decline in the incidence of amyloidosis secondary to tuberculosis has been largely due to the introduction of effective anti-TB therapy and clear diminution in TB rate.^11^ In one study, the postmortem incidence of amyloidosis declined from 24% in the prechemotherapy years to 11% after the introduction of streptomycin and isoniazid.^12^ Tuberculosis still remains the commonest cause of secondary renal amyloidosis in developing countries including India.^13^–^17^

The interval between the onset of predisposing disease and first evidence of amyloidosis is variable in different studies. This interval was reported to be between 6 months to 43 years (mean 17 years) by Kennedy *et al*^8^, 2 months to 31 years by Mehta *et al*,^16^ 5 months to 25 years by Shah *et al*,^17^ 1–30 years (mean 6.92 years) by Chug *et al*,^15^ 1–6 years (mean 2 years) by Erk *et al*,^18^ and 2 months to 6 years by Gupta *et al*.^19^ In the present study this interval was 2 months to 7 years (mean 2.25 years). It is often believed that secondary amyloidosis occurs months to years after the onset of the predisposing cause. In our study 15.1% patients were detected to have amyloidosis in less than three months period after the diagnosis of PTB. A very early onset amyloidosis after the diagnosis of active TB has also been reported by El-Hennawy *et al*^20^ and Malhotra *et al*,^21^ in recent years. Since the clinical onset of amyloidosis is preceded by a variable preclinical stage, the true interval between the preceding disease and the onset of amyloidosis is not known exactly. Therefore, we believe it is reasonable to suspect renal amyloidosis in any patient with known history of PTB presenting with pedal edema and proteinuria.

Proteinuria is most consistent feature of renal amyloidosis. It may be moderate but is generally abundant. The reported incidence of this complication varied from 32–68%.^15^ All patients in the present study showed variable proteinuria, and it was heavy with 24-hour protein excretion of >10 g in 15 (35%) patients. The nephrotic syndrome occurs frequently but not constantly as observed in 64% cases in one study.^22^ In our study it was seen in 23 (53.4%) cases. The absence of nephrotic syndrome is not necessarily linked with slight proteinuria and patients with normal serum albumin may have heavy proteinuria, this apparent paradox was earlier reported by Heptinstal.^23^ Hypertension is also variable in renal amyloidosis patients with reported incidence of 7–50%.^15^,^22^ Nineteen out of forty three patients (44%) in the present series were hypertensive.

All patients with PTB and renal amyloidosis in our study had pedal edema with or without generalized puffiness, renal medical disease on ultrasound abdomen, and proteinuria, suggesting that these features should arouse suspicion of amyloidosis in any TB patient and consideration of renal biopsy for confirmation.

It has been postulated that the process of amyloid formation may be a nucleation-initiated event, influenced by local tissue factors, including macrophage activity. Once the nidus is formed, it may accelerate deposition of larger numbers of AA fibrils.^24^ Such a theory would explain the increased propensity for amyloid accumulation in some of our cases having relapse of PTB. Since persistent inflammation is continuous source of SAA with subsequent amyloid deposition in the kidneys with other factors, this mechanism also explains increased risk of renal amyloidosis in patients having extensive PTB – moderately advanced/far advanced/destroyed lung disease – where permanent structural damage causes persistent inflammation despite adequate anti-TB therapy. In a study of 40 patients with renal amyloidosis, TB was considered active in only two patients at the time of diagnosis of amyloid disease.^8^ In another study, 9–10% of all patients with various stages of PTB despite effective anti-TB therapy eventually developed proteinuria due to renal amyloidosis after a certain period of time. It has also been postulated that once amyloidosis has extensively involved the kidneys, anti-TB treatment will not cause any regression in the course of renal amyloidosis.^25^ Fifteen adequately treated patients of PTB in our study also developed renal amyloidosis despite adequate treatment.

A prolonged course with apparent remissions has occasionally been reported, but is certainly exceptional. Most patients with renal amyloidosis ultimately progress to chronic renal failure. Few instances of apparent remission of amyloid disease have been published with improvement in general condition and disappearance of nephrotic syndrome,^22^,^26^ However, to the best of our knowledge, there has never been histological evidence of disappearance of amyloidosis.

There were some limitations in the present study. In 24 patients of TB where the kidney tissue was not obtained on renal biopsy, we could not do repeat renal biopsy or search for amyloid deposition on rectal mucosa or liver biopsy in patients with clinical evidence of renal involvement. Such an approach would have increased the number of patients for better evaluation. Further, we could not study the effect of anti-TB therapy on the reversal of renal amyloidosis in new patients of TB because most patients were irregular on their followup, while others refused repeat renal biopsy.

In conclusion, despite limitations, the present study explores few important features of coexistent PTB and renal amyloidosis. It is suggested that presence of pedal edema, proteinuria, and grossly abnormal kidneys on USG in TB patients, especially those with extensive lesions on chest radiography, should be evaluated for amyloidosis by renal biopsy. Further, adequately treated patients may present with renal amyloidosis despite having effective anti-TB therapy and this could be attributed to post tubercular bronchiectasis or an irreversible process of amyloid deposition that had initiated earlier. The onset of amyloidosis may be early, rather than many years. This is contrary to general belief that renal amyloidosis develops only in chronic cases of PTB with duration of illness often ten or more years. An increase in awareness of the clinical features of amyloidosis in TB is important for its early diagnosis and timely assessment.
